# Impacts of Activation of the Mitogen-Activated Protein Kinase Pathway in Pancreatic Cancer

**DOI:** 10.3389/fonc.2015.00023

**Published:** 2015-02-04

**Authors:** Toru Furukawa

**Affiliations:** ^1^Institute for Integrated Medical Sciences, Tokyo Women’s Medical University, Tokyo, Japan

**Keywords:** pancreatic cancer, mitogen-activated protein kinase, KRAS, BRAF, DUSP6, ERK, MKP-3, microRNA

## Abstract

Pancreatic cancer is characterized by constitutive activation of the mitogen-activated protein kinase (MAPK) pathway. Mutations of *KRAS* or *BRAF* and epigenetic abrogation of DUSP6 contribute synergistically to the constitutive activation of MAPK. Active MAPK induces the expression of a variety of genes that are thought to play roles in malignant phenotypes of pancreatic cancer. By blocking the functions of such induced genes, it is possible to attenuate the malignant phenotypes. The development of drugs targeting genes downstream of MAPK may provide a novel therapeutic option for pancreatic cancer.

## Introduction

Pancreatic cancer is one of the leading causes of cancer mortality in Japan and many western countries (1, 2). Despite improvements in diagnostic and therapeutic modalities, the 5-year survival rate of patients with pancreatic cancer is still <10% (3). This poor prognosis elicits a pressing need for the development of effective diagnostic and therapeutic measures to improve patient survival. An understanding of the molecular pathobiology involved may provide clues that ultimately lead to the fulfillment of such needs. Pancreatic cancer is characterized by constitutive activation of the mitogen-activated protein kinase (MAPK) pathway. The purpose of this review is to explore the mechanisms and relevance of MAPK activation in the pathobiology of pancreatic cancer.

## RAS–MAPK Pathway

**Figure 1:**
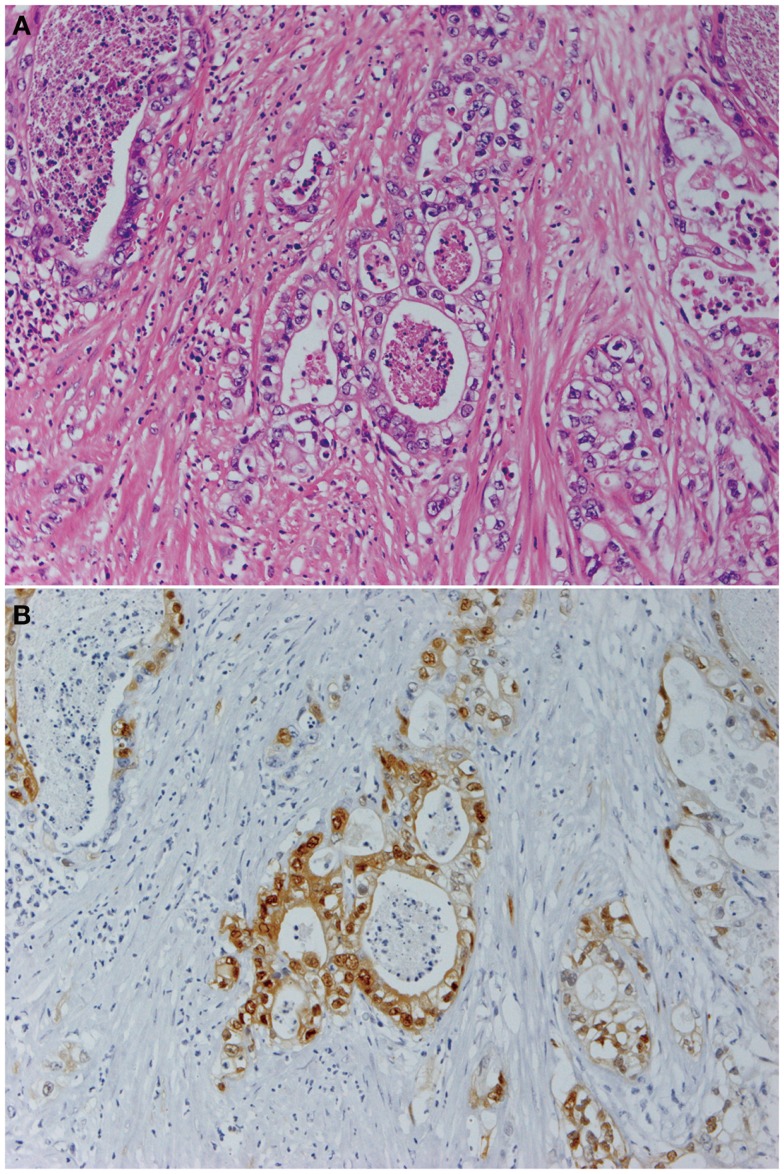
**Pancreatic cancer produces constitutively active MAPK**. Panels show histological images of invasive ductal adenocarcinoma **(A)** and its expression of phosphorylated MAPK **(B)**. Staining with hematoxylin and eosin **(A)** and indirect immunohistochemistry using anti-phosphorylated MAPK antibody (137F5, 1:250; Cell Signaling Technology Inc., Danvers, MA, USA) and a Histofine SAB-PO kit (Nichirei Corp., Tokyo, Japan) according to the manufacturer’s instructions **(B)**. Original magnification was ×200.

The vast majority of pancreatic cancers involve gain-of-function mutations in *KRAS* (4). *KRAS* encodes RAS, a guanosine triphosphate (GTP)-binding protein that, when activated by binding with GTP, triggers stimulation of various downstream signaling pathways (5). The activation of RAS is mediated by a guanine nucleotide exchange factor, which is activated by upstream ligand-binding receptor tyrosine kinases (5). Active RAS is self-inactivated by intrinsic GTPase activity, with the help of a GTPase-activating protein (5). Mutations of *KRAS* observed in pancreatic cancers often occur in codons 12, 13, and 61, at most are G12D or G12R substitutions, which attenuates the intrinsic GTPase activity, thus, such mutations result in prolonged activation of RAS (6). Moreover, some pancreatic cancers harbor activating mutations of *BRAF* rather than *KRAS* (7). *BRAF* encodes B-RAF, a serine/threonine kinase belonging to a family of mitogen-activated protein kinase kinase kinases (MAP3Ks). Mutations in *BRAF* commonly involve codon 600 and often result in a V600E substitution, which leads to constitutive activation of its kinase function (8). Mutations of *KRAS* and *BRAF* are mutually exclusive in pancreatic cancers, which suggest that the activating mutations of these genes can compensate for each other in pancreatic cancer phenotypes (7). B-RAF is an immediately downstream of RAS and activates MAP2K1/MEK, which in turn activates MAPK1/ERK2. Thus, the activating signal is passed on by a chain of kinase reactions, which forms MAPK signaling pathway. Hence, activating mutations of *KRAS* and *BRAF* ultimately result in activation of the MAPK signaling pathway, which is crucial for pancreatic cancer (Figure 1). MAPKs are classified into three classes according to their distinctive effectors, ERKs, JNKs, and p38MAPKs (9). Each class of MAPK is involved in different functions, i.e., ERKs are involved mainly in mitosis and proliferation, JNKs in apoptosis and differentiation, and p38MAPKs in stress responses. Activated MAPKs translocate into the nucleus and phosphorylate a variety of transcription factors, altering the expression of various genes, and therefore enacting specific cellular responses (10).

## Dual-Specificity Protein Phosphatases

Dual-specificity protein phosphatases dephosphorylate phosphorylated serine, threonine, or tyrosine residues of substrates. Dual-specificity protein phosphatases (DUSPs) share a common structure that comprises a carboxyl-terminal catalytic domain and an amino-terminal non-catalytic domain. The catalytic domain contains a sequence homologous to the prototypic VH-1 DUSP encoded by the vaccinia virus (11, 12). The non-catalytic domain contains both a conserved cluster of basic amino acid residues involved in specific recognition of MAPKs, known as the kinase interaction motif, and sequences that determine the subcellular localization of these enzymes (12, 13). MAPKs are prime substrates for DUSPs, i.e., DUSPs primarily dephosphorylate target residues in MAPKs and induce their inactivation. DUSPs are classified into three classes by their subcellular localization and substrate specificities (12). The first class includes DUSP1, DUSP2, DUSP4, and DUSP5, which act in the nucleus and have broad specificities toward various MAPKs. The second class includes DUSP6, DUSP7, and DUSP9, which act in the cytoplasm and strictly on MAPK1/ERK2 (13). The last class includes DUSP8, DUSP10, and DUSP16, which act mainly on MAPK8/JNK1 and MAPK14/p38. DUSPs and MAPKs form feedback loops, each controlling the activities of the other.

## DUSP6 in Pancreatic Cancer

Owing to its uniquely strict specificity toward MAPK1/ERK2 whose activation is involved in oncogenesis, an association between DUSP6 and cancer has been a prime focus of study. DUSP6 resides on the chromosome locus 12q21–22, which is commonly deleted in pancreatic cancer (14). In studies of genetic and epigenetic alterations of *DUSP6* in pancreatic cancer, mutations of this gene have not been observed, however, marked reduction of its expression has been found (15). This reduced expression is associated with constitutive activation of MAPK1/ERK2 and, despite the presence of mutated *KRAS*, exogenous overexpression of DUSP6 induces dephosphorylation of MAPK1/ERK2, subsequent suppression of proliferation, and eventual apoptosis of pancreatic cancer cells (16). This indicates that, for their proliferation and survival, pancreatic cancer cells are largely dependent on the activation of MAPK induced as a consequence of the synergistic effect of mutations in *KRAS* and an abrogation of DUSP6.

While the expression of DUSP6 is markedly reduced in pancreatic cancer cells, the expression of DUSP6 can be restored by treatment with 5-azacytidine, an inhibitor of DNA methyltransferase, which indicates that the reduced expression stems from hypermethylation. A search for possible genomic regions of hypermethylation pertaining to the reduced expression of DUSP6 resulted in the discovery of a cluster of methylated CpG in intron 1 of *DUSP6* gene (17). A promoter assay indeed showed promoter activity of the intron 1 (18). Moreover, this activity is shown to be positively associated with activity of MAPK1/ERK2. In other words, active MAPK1/ERK2 induces upregulation of the promoter activity of *DUSP6*, resulting in an increase in its expression. The increased expression of DUSP6 leads to inactivation of MAPK1/ERK2, hence, which indicates that a negative feedback loop is formed between these two molecules (18).

Pancreatic cancer is hypothesized to develop through multistep progression of dysplastic grades of epithelial cells in the pancreatic duct along with accumulation of genetic abnormalities, which is known as the progression model of pancreatic cancer (19). In this progression model, low-grade dysplasia, termed low-grade pancreatic intraepithelial neoplasia (PanIN), is thought to progress into high-grade dysplasia, or high-grade PanIN, and eventually into invasive carcinoma. The progression is associated with an accumulation of genetic aberrations in *KRAS*, *CDKN2A*, *SMAD4*, and *TP53* (19, 20). Therefore, PanIN is regarded as a precursor lesion for pancreatic cancer. The expression of DUSP6 is increased in PanINs while it is decreased in invasive carcinoma, which indicates that DUSP6 may be a gatekeeper preventing progression of the precursor lesion into a fully invasive cancer (21).

DUSP6 is also known to acquire post-translational control of its expression. DUSP6 is degraded by the ubiquitin–proteasome system. For such degradation to take place, serines 159 and 197 of DUSP6 need to be phosphorylated by MAPK1/ERK2 (22). This phosphorylation event is regarded as a positive feedback mechanism for the activation of MAPK1/ERK2 (22). The reduced expression of DUSP6 in cancer cells might be due to the acceleration of this degradation system, which is indeed the case in ovarian cancer cells. The accelerated degradation of DUSP6 in these cells is associated with reactive oxygen species, therefore, blockage of which by an antioxidant increases DUSP6 expression, dephosphorylation of MAPK1/ERK2, and downregulation of cell proliferation (23).

## Effects of MAPK Activation

Phosphorylated MAPK1/ERK2 translocates into the nucleus, phosphorylates various transcription factors, and induces expression of downstream genes that are thought to play significant roles in malignant phenotypes of pancreatic cancer (10, 24). To identify such downstream genes, a comparison of the gene expression profiles of pancreatic cancer cells with and without exogenous expression of DUSP6, which dephosphorylates MAPK, was carried out (24). The comparison revealed numerous differentially expressed genes involved in the cell cycle and mitosis, DNA replication, receptor signaling, and transport. These included *AURKA, AURKB, TPX2, CENPA, EPHA2, CCNB2, IFITM1, RARRES3, SOX4*, and *PYCARD* (24).

*AURKA* encodes aurora kinase A, a kinase that phosphorylates molecules involved in spindle formation during mitosis (25). Promoter activity of *AURKA* is positively associated with MAPK activity, i.e., active MAPK upregulates the promoter activity and induces the expression of *AURKA* (24). The promoter region of *AURKA* harbors binding sites for ETS2, a substrate of MAPK (26). Knockdown of ETS2 induces downregulation of *AURKA* (24). These results indicate that active MAPK induces the expression of *AURKA* via ETS2. Aurora kinase A is overexpressed in the majority of pancreatic cancers, which is consistent with the observation that most pancreatic cancers produce constitutively active MAPK. Knockdown of *AURKA* in pancreatic cancer cells results in cell cycle arrest at G2/M and hence, suppression of cellular proliferation (27). Moreover, the knockdown of *AURKA* enhances the effect of chemotherapy with taxane (27).

To identify genes with an important role in cellular proliferation from among those downstream of MAPK, a knockdown screen was conducted, testing the effect of RNA interference targeting the downstream genes on *in vitro* proliferation of pancreatic cancer cells (28). The results of this study indicated that *AURKB*, *CENPA, EBNA1BP2, GOLT1A, KIF11, NEDD4L, SON, TPX2*, and *WDR5* play a crucial role in *in vitro* proliferation, similarly to *AURKA* (28). Among them, *SON*, a gene encoding SON, which is involved in nuclear speckle organization and RNA processing, was the most significant target for suppression of proliferation (28). SON is required for efficient RNA processing of transcripts involved in cell cycle progression (29, 30). Knockdown of *SON* in cultured pancreatic cancer cells caused G2/M arrest and apoptosis (28). By using a subcutaneous xenograft model in immunocompromised mice, pancreatic cancer cells with knockdown of SON resulted in a significant reduction in *in vivo* tumorigenesis (28). Moreover, SON is overexpressed in ductal adenocarcinoma of the pancreas, while its expression is lower in PanIN and normal ductal cells (28). The suppressive effect of SON knockdown on *in vitro* proliferation is unremarkable in normal ductal cells (28). These results indicate that pancreatic cancer cells may depend on SON to maintain their active proliferation and tumorigenicity (28). Effectiveness of these targetable downstream genes for treatment of various cancers has been also reported recently (31–37).

MicroRNAs are endogenous short non-coding RNAs that interact with protein-coding messenger RNAs and regulate translation (38, 39). MiRNAs play important roles in physiological development as well as in pathological conditions, including cancer (40). They are aberrantly expressed in many cancers including pancreatic cancer. Such aberrant expression is thought to be primarily associated with an altered copy number of genomic loci coding for miRNAs. However, signal transduction pathways can also alter the expression of miRNAs, some of which therefore may be affected by active MAPKs in pancreatic cancer. The association between miRNAs and MAPK in pancreatic cancer cells was investigated by comparing miRNA expression in cells with active or attenuated MAPK (41). The results indicated that miR-7, miR-34a, miR-193b, and miR-181d were preferentially associated with MAPK activity, of which, miR-7 was upregulated, while miR-34a, miR-193b, and miR-181d were downregulated. MiR-7 is known to target *EGFR*, a gene upstream of MAPK. The upregulation of miR-7 by MAPK may induce downregulation of EGFR and subsequent downregulation of MAPK, thus forming a feedback loop. MiR-34a is a highly conserved miRNA and is known to be upregulated by tumor protein 53 (TP53), a tumor suppressor (42, 43). MAPK downregulates miR-34a, therefore, MAPK is thought to counteract TP53’s role in controlling the expression of this miRNA, and can attenuate TP53’s tumor suppressive function. Expression of miR-193b is lowered in various cancers, and its overexpression induces suppression of proliferation of cancer cells, including those of pancreatic cancer. Targets of miR-193b have been shown to be *CCND1*, *NT5E*, *PLAU*, *STARD7*, *STMN1*, and *YWHAZ*, which are involved in the cell cycle, conversion of adenosine monophosphate to adenosine, urokinase-type plasminogen activation, lipid binding, cell cycle progression, and signal transduction, respectively (41). Some of these genes are shown to promote proliferation of pancreatic cancer cells, such that suppression of miR-193b by MAPK would result in accelerated proliferation. Associations between MAPK and miRNAs and their relevance for diseases have been also reported (44–47).

## Conclusion and Perspectives

**Figure 2:**
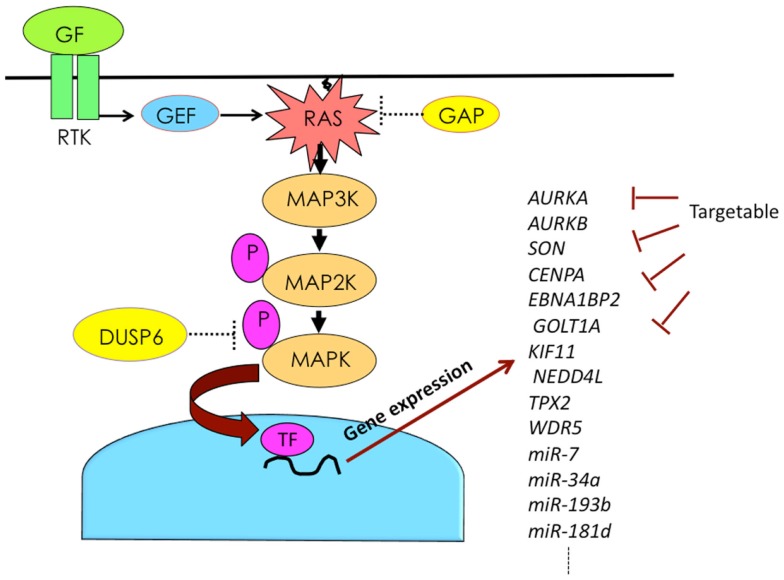
**Activation of RAS–MAPK pathway induces expression of numerous genes, some of which can be targeted**. Abbreviations are GAP, GTPase-activating protein; GEF, guanine nucleotide exchange factor; GF, growth factor; P, phosphorylation; RTK, receptor tyrosine kinase; and TF, transcription factor.

The combination of *KRAS* mutation and DUSP6 abrogation plays a synergistic role in the constitutive activation of MAPK in pancreatic cancer. Active MAPK induces a variety of genes, including miRNAs involved in malignant cancer phenotypes. By blocking the functions of such induced genes, it is possible to attenuate malignant phenotypes (Figure 2). The development of drugs targeting genes downstream of MAPK may provide a novel therapeutic option for pancreatic cancer.

## Conflict of Interest Statement

The author declares that the research was conducted in the absence of any commercial or financial relationships that could be construed as a potential conflict of interest.
